# The effect of cycloplegia in the accuracy of autorefraction, keratometry and axial length using the Myopia Master

**DOI:** 10.1186/s12886-024-03529-z

**Published:** 2024-08-01

**Authors:** Agustin Peñaranda, Oscar Torrado, Ana Márquez, António M. Baptista, Pedro Miguel Serra

**Affiliations:** 1 Ophthalmology Clinic Vista Sánchez Trancón, Vista Sánchez Trancón Building Tecnolaser, Room 14 Calle La Violeta, Badajoz, 06005 Spain; 2 Centre of Physics, University of Minho, Braga, Portugal

**Keywords:** Refractive error, Biometry, Repeatability, Agreement, Axial length, Cycloplegia

## Abstract

**Background:**

Assessing refractive errors under cycloplegia is recommended for paediatric patients; however, this may not always be feasible. In these situations, refraction has to rely on measurements made under active accommodation which may increase measurements variability and error. Therefore, evaluating the accuracy and precision of non-cycloplegic refraction and biometric measurements is clinically relevant. The Myopia Master, a novel instrument combining autorefraction and biometry, is designed for monitoring refractive error and ocular biometry in myopia management. This study assessed its repeatability and agreement for autorefraction and biometric measurements pre- and post-cycloplegia.

**Methods:**

A prospective cross-sectional study evaluated a cohort of 96 paediatric patients that underwent ophthalmologic examination. An optometrist performed two repeated measurements of autorefraction and biometry pre- and post-cycloplegia. Test-retest repeatability (TRT) was assessed as differences between consecutive measurements and agreement as differences between post- and pre-cycloplegia measurements, for spherical equivalent (SE), refractive and keratometric J0/J45 astigmatic components, mean keratometry (Km) and axial length (AL).

**Results:**

Cycloplegia significantly improved the SE repeatability (TRT, pre-cyclo: 0.65 D, post-cyclo: 0.31 D). SE measurements were more repeatable in myopes and emmetropes compared to hyperopes. Keratometry (Km) repeatability did not change with cycloplegia (TRT, pre-cyclo: 0.25 D, post-cyclo:0.27 D) and AL repeatability improved marginally (TRT, pre-cyclo: 0.14 mm, post-cyclo: 0.09 mm). Regarding pre- and post-cycloplegia agreement, SE became more positive by + 0.79 D, varying with refractive error. Myopic eyes showed a mean difference of + 0.31 D, while hyperopes differed by + 1.57 D. Mean keratometry, refractive and keratometric J0/J45 and AL showed no clinically significant differences.

**Conclusions:**

Refractive error measurements, using the Myopia Master were 2.5x less precise pre-cycloplegia than post-cycloplegia. Accuracy of pre-cycloplegic refractive error measurements was often larger than the clinically significant threshold (0.25 D) and was refractive error dependent. The higher precision compared to autorefraction measurements, pre- and post-cycloplegia agreement and refractive error independence of AL measurements emphasize the superiority of AL in refractive error monitoring.

## Introduction

Refractive error measurements obtained through autorefraction, whether in non-cycloplegic or cycloplegic conditions, play a crucial role in evaluating an individual’s refractive status. The comparison between non-cycloplegic and cycloplegic autorefraction is essential for understanding the eye accommodative behaviour, particularly in paediatric or non-collaborative cases. In epidemiologic visual screenings, accurate and repeatable non-cycloplegic measurements are vital to prevent mischaracterization of the population’s refractive status and to avoid false-negative cases requiring further clinical attention [1].

With the increasing global prevalence of myopia [2] and its association with eye-elongation [3], modern autorefractors-keratometers, such as the Myopia Master (OCULUS Optikgeräte GmbH, Wetzlar, Germany) have incorporated axial length measurement systems. These instruments enable axial length growth monitorization throughout time and may be regarded as a more accurate method for refractive error monitoring, since it minimizes the effects of accommodation. Compared to cycloplegic manifest refraction the Myopia Master’s cycloplegic refraction shows a more negative (∼ -0.50 D) spherical equivalent (SE) [4]. In contrast to other table-top autorefractors systematic SE differences typically range from 0.4 to ∼0.0 D [4–6]. Regarding biometric measurements, comparisons between the Myopia Master and the gold-standard biometer (IOL Master 700), reveal systematic differences in the range of 0.02 to 0.2 D for mean keratometry (Km) and − 0.004 to 0.036 mm for axial length (AL) [4–7]. However, these discrepancies fall below clinically significant levels.

Yet, the repeatability and accuracy of non-cycloplegic and cycloplegic autorefraction and biometry with the Myopia Master remain unreported. These features are contingent upon the instrument ability to mitigate proximal accommodation and to its underlying operational principles [8, 9]. This is particularly relevant when cycloplegic refraction is not performed or cycloplegia is legally restricted [10]. Additionally, the administration of cycloplegic drugs induces anatomical changes, notably in the crystalline lens’s shape and dynamics [11], which can influence the repeatability and agreement of AL measurements [12].

This study aimed to evaluate the repeatability of autorefraction, keratometry, and axial length measurements, both non-cycloplegic and cycloplegic, using the Myopia Master in a paediatric population. Additionally, it assessed the agreement between non-cycloplegic and cycloplegic measurements, addressing the limited existing studies involving the Myopia Master in the paediatric population.

## Methods

### Participants

This prospective cross-sectional study enrolled 96 paediatric patients, aged under 16 undergoing routine ophthalmological evaluation at the Ophthalmology Clinic Vista Sánchez Trancón, Badajoz, Spain. Inclusion criteria encompassed refractive astigmatism below 2.50 D under cycloplegia, distance corrected visual acuity (DCVA) equal to or better than 6/6, absence of ocular pathology and strabismus. The Myopia Master measurement protocol, utilizing the “Myopia Mode”, performed autorefraction, keratometry and AL measurement sequentially. Only measurements with a quality index ≥ 7 for autorefraction and keratometry, and a signal-to-noise ratio ≥ 6.0 for AL were included. For this study, only measurements from the right eyes were included. The study adhered to the Helsinki Declaration principles and received approval from the local ethics committee (Comité Ético para Investigacion Clinica de Badajoz, Spain). Patient information was provided to the accompanying caregiver and informed consent was obtained from all participants.

### Study protocol

Myopia Master measurements were integral to the ophthalmological examination, which included visual acuity assessment, autorefraction, keratometry, AL measurement, subjective refraction pre- and post-cycloplegia, cover test, slit-lamp examination and ophthalmoscopy. Cycloplegia was induced using Cyclopentolate (Colicusí Cicloplégico 10 mg/ml, Alcon), with a first drop followed by a second one ten minutes later. Cycloplegic measurements with the Myopia Master were taken 30 min after the initial drop. Both pre- and post-cycloplegic measurements were repeated within five-minute intervals to evaluate intrasession repeatability. Patients were instructed to keep both eyes open, blink naturally, and fixate on the centre of a hot-air balloon used as fixation target. The administration of cycloplegic drops and measurements were consistently performed by the same senior optometrist (AP).

### Instrument – Myopia Master

The Myopia Master (version 7.2 R3) was utilized for autorefraction, keratometry, and AL measurements. It incorporates a fixation target simulating optical infinity coupled with a fogging system for accommodation control during autorefraction. The operational principles of the keratometer and biometer were previously detailed [7]. The autorefractor employs an infrared light source (λ = 850 nm) projecting light onto the retina. A charge-coupled device camera captures the reflected light, recording its deviation from the shutter location. An integrated micro-computer calculates the ametropia by converting the infrared light to visible light (λ = 546.1 nm, Oculus Spain personal communication), and the reported autorefraction reflects the average of three measurements.

### Statistical analysis

Refractive and biometric parameters (totalling seven) were analysed for measurement repeatability, both pre- and post-cycloplegia, and for measurement agreement between pre- and post-cycloplegia. Refractive parameters included SE in dioptres (D) and refractive astigmatic vector components (J_0__Ar and J_45__Ar in D). Biometric parameters included Km (in D), keratometric astigmatic vector components (J_0__K and J_45__K in D), and AL (in mm). Spherical equivalent was calculated as sphere plus half of the refractive cylinder in negative power (SE = sphere + Cylinder/2). The J_0_ vector component represents the Jackson-cross cylinder power at 180 and 90 degrees, while J_45_ denotes the Jackson-cross cylinder power at 45 and 135 degrees. The J_0_ and J_45_ components, whether refractive or keratometric, were computed using the formulas J_0_= -Cylinder/2 × cos(2× Cylinder axis) and J_45_ = -Cylinder/2 × sin(2× Cylinder axis), where the cylinder axis indicates the orientation of the most powerful meridian [13].

Data are presented as mean, standard deviation (± SD), 95% confidence interval (CI) for the mean and range. The Kolmogorov-Smirnov test was used for assessing data distribution. Paired comparisons were performed using the Student’s t-test for normally distributed data and Wilcoxon matched-pairs test for non-normally distributed data. Statistical significance was adjusted using Bonferroni criterium (0.05/number of comparisons, 0.05/7 = 0.007).

Measurements’ repeatability, defined as the test-retest repeatability (TRT), was calculated as 1.96× average standard deviation (SD) of the differences between repeated measurements. The TRT indicates the range within which 95% of the differences between measurements fall. The 95% confidence interval of the TRT, representing the interval encompassing the true dispersion of differences for the population, was determined using the chi-squared (χ²) distribution [14].

Measurements’ agreement was calculated as the difference between average post-cycloplegia and average pre-cycloplegia measurements. The 95% limits of agreement (LoA) were estimated as 1.96× average SD of the difference between pairs of measurements. The 95% CI of the LoA representing the true dispersion of the LoA were calculated using exact methods [15].

Repeatability and agreement were further analysed based on cycloplegic refractive error (Myopes ≤-0.75 D; Emmetropes>-0.75 D and < + 1.00 D; Hyperopes ≥ + 1.00 D) [16]. Group comparisons were made using one-factor analysis of variance (ANOVA) or non-parametric Kruskall-Wallis according to the normality of the data. Pairwise comparisons p-value was adjusted using Bonferroni criterium (*p* = 0.05/3 = 0.017). The sample size calculated using the SE parameter for detecting a difference of 0.25D between pre- and post-cycloplegia SE with a standard deviation of 0.85 D and a power of 0.80 was 93 patients, assuming a type error I probability of 0.05. Statistical analysis was conducted using IBM SPSS v23.

## Results

Table 1 shows autorefraction and biometric parameters data for 96 patients (Females: 48 |Males: 48) measured pre- and post-cycloplegia. The sample, with a mean age of 12.5 ± 2.4 years-old (range 7; 16), included 35 myopes SE= -2.40 ± 1.23 D (range: -5.64; -0.79), 30 emmetropes SE = + 0.31 ± 0.48 D (range: -0.66; +0.95) and 31 hyperopes SE = + 2.54 ± 1.62 D (range: +1.00; +7.25).

**Table 1:** Autorefraction, keratometry and axial length measured pre- and post-cycloplegia. Spherical equivalent (SE), refractive astigmatism vectorial components (J0_Ar and J45_Ar), mean keratometry (Km), corneal astigmatism vectorial components (J0_K and J45_K), and axial length (AL). Data is presented with mean ± standard deviation (SD), 95% confidence interval (CI) and range

	Measurement 1			Measurement 2		
Parameter	Mean ± SD	95% CI	Range	Mean ± SD	95% CI	Range
**Pre-cycloplegia**						
SE (D)	-0.80 ± 2.04	-1.21; -0.39	-6.20; +6.51	-0.81 ± 1.99	-1.21; -0.40	-6.35; +5.18
J_0__Ar (D)	+ 0.20 ± 0.36	+ 0.12; +0.27	-0.78; +1.15	+ 0.21 ± 0.37	+ 0.14; +0.29	-0.78; +1.23
J_45__Ar (D)	-0.08 ± 0.20	-0.12; -0.04	-0.75; +0.60	-0.09 ± 0.19	-0.13; -0.05	-0.47; +0.53
Km (D)	43.40 ± 1.54	43.09; 43.71	39.85; 47.45	43.39 ± 1.53	43.08; 43.70	39.90; 47.45
J_0__K (D)	+ 0.34 ± 0.24	+ 0.29; +0.39	-0.31; +0.94	+ 0.33 ± 0.23	+ 0.28; +0.38	-0.28; +0.94
J_45__K (D)	+ 0.01 ± 0.13	-0.02; +0.04	-0.35; +0.35	+ 0.01 ± 0.13	-0.02; +0.04	-0.35; +0.35
AL (mm)	23.41 ± 1.23	23.16; 23.66	20.42; 26.68	23.41 ± 1.22	23.17; 23.66	20.43; 26.67
**Post-cycloplegia**						
SE (D)	-0.02 ± 2.39	-0.50; +0.47	-5.61; +7.18	-0.01 ± 2.39	-0.49; +0.47	-5.68; +7.32
J_0__Ar (D)	+ 0.25 ± 0.33	+ 0.18; +0.32	-0.60; +1.19	+ 0.24 ± 0.35	+ 0.20; +0.34	-0.66; +1.19
J_45__Ar (D)	-0.07 ± 0.20	-0.11; -0.03	-0.76; +0.60	-0.08 ± 0.19	-0.12; -0.04	-0.50; +0.60
Km (D)	43.38 ± 1.57	43.07; 43.70	39.85; 47.55	43.37 ± 1.55	43.06; 43.68	39.60; 47.35
J_0__K (D)	+ 0.34 ± 0.24	+ 0.29; +0.39	-0.29; +0.91	+ 0.35 ± 0.24	+ 0.30; +0.39	-0.28; +0.89
J_45__K (D)	+ 0.02 ± 0.13	-0.01; +0.05	-0.24; +0.45	+ 0.02 ± 0.13	-0.01; +0.05	-0.24; +0.45
AL (mm)	23.42 ± 1.20	23.18; 23.67	20.42; 26.68	23.42 ± 1.20	23.17; 23.67	20.43; 26.67

### Autorefraction

**Fig. 1:**
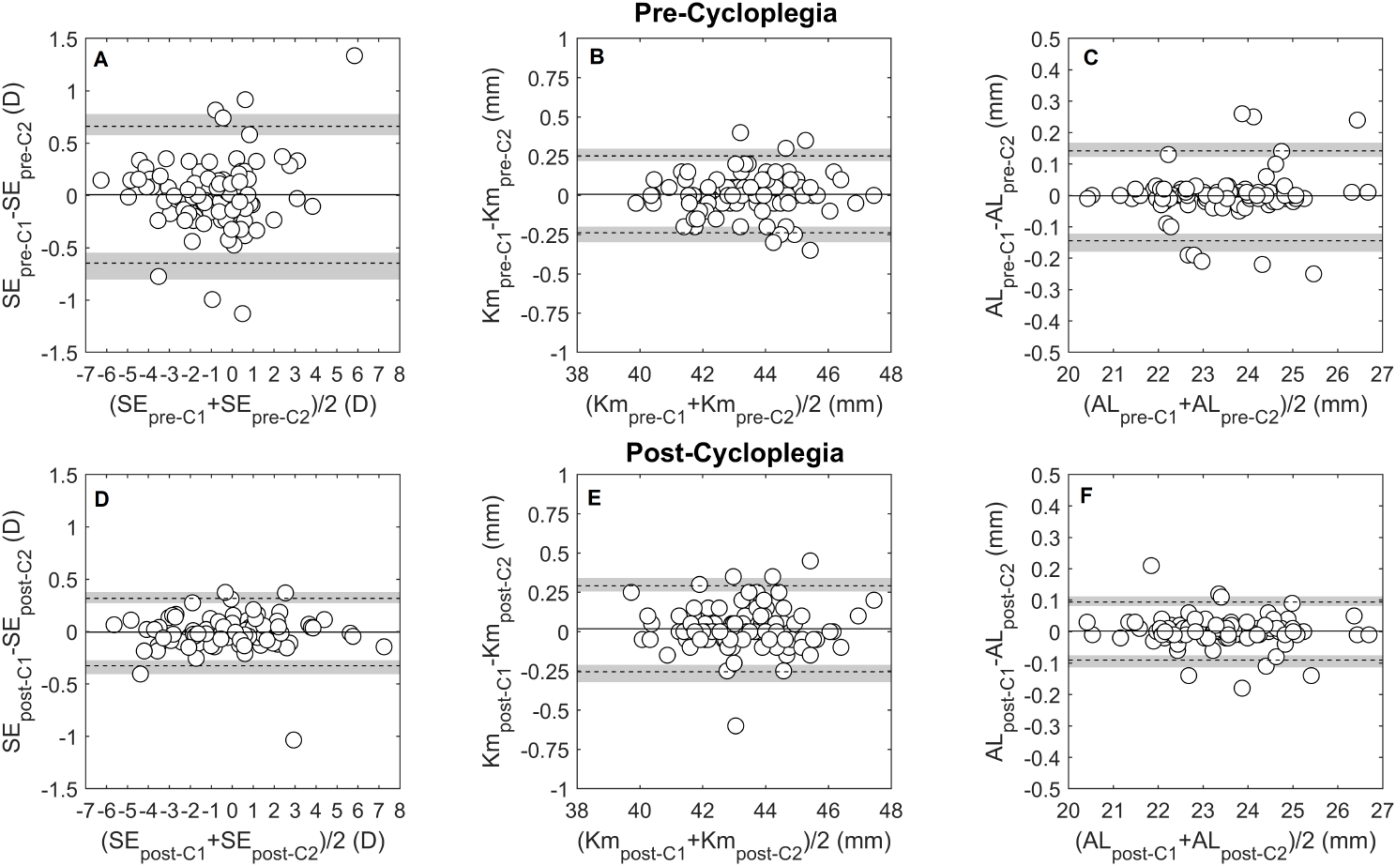
Bland-Altman plots for the intrasession repeatability pre- (**A** to **C**) and post-cycloplegia (**D** to **F**), for the following parameters: spherical equivalent (SE in D), mean keratometry (Km in D) and axial length (AL in mm). Continuous lines in the figures indicate the mean difference between measurement and repeated measurement, dashed lines the 95% limits of repeatability and the grey lines represent the 95% confidence interval of the limits of repeatability

**Fig. 2:**
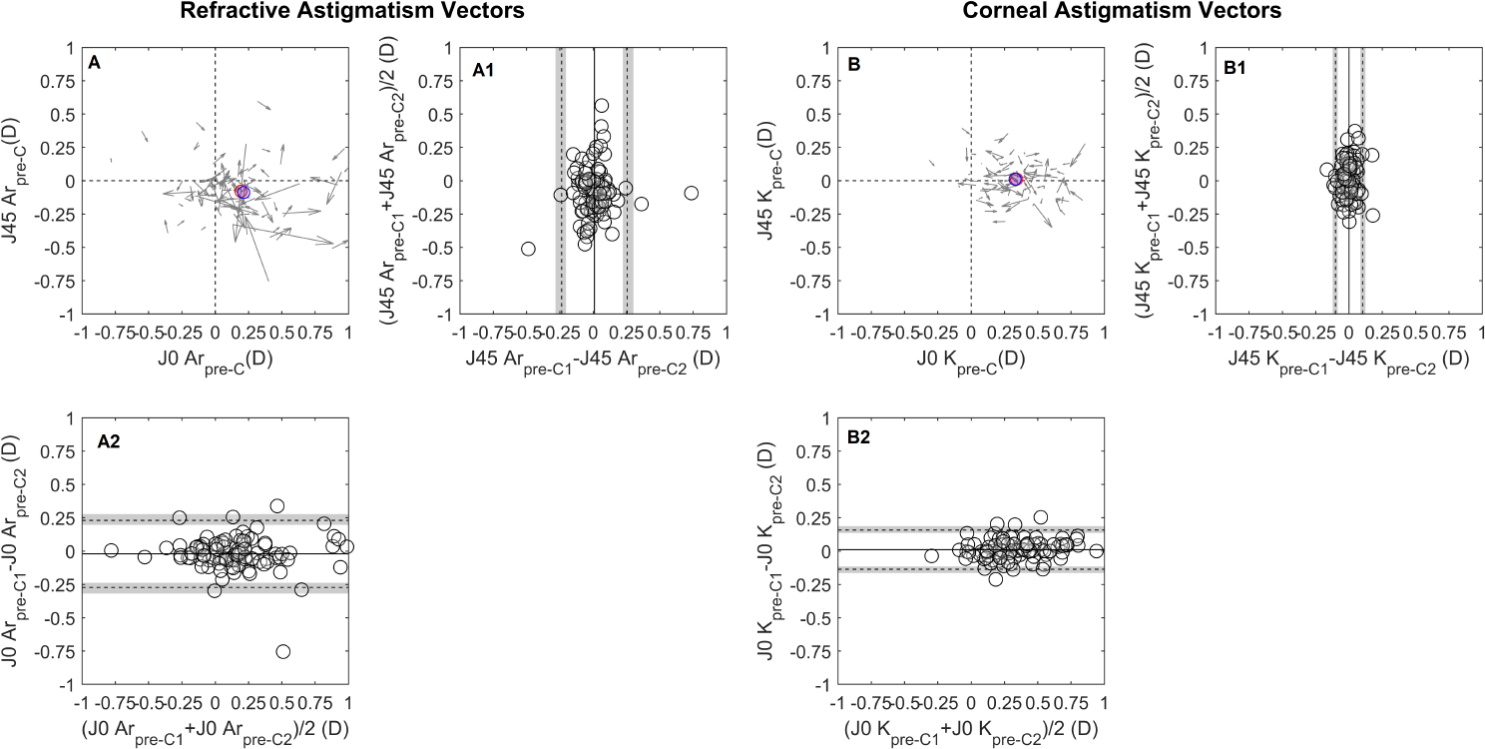
Bland-Altman (B-A) plots for the intrasession repeatability pre-cycloplegia, (A) refractive and (B) corneal astigmatism vectorial components (J0/J45). Cartesian plots (A and B) show the J0/45 variation observed during the test-retest (the arrow starting point indicates measurement 1 J0 and J45 coordinates and the ending represents measurement 2. The J0 and J45 B-A plots are presented below and laterally to the cartesian plot. The middle point of each vector read on the x-axis and y-axis corresponds to a data point in the x -axis of the J0 B-A and y-axis of the J45 B-A plot. The details of the B-A plots are as in Fig. 1. The red and blue circles represent the mean J0/J45 for measurement 1 and measurement 2, respectively

**Fig. 3:**
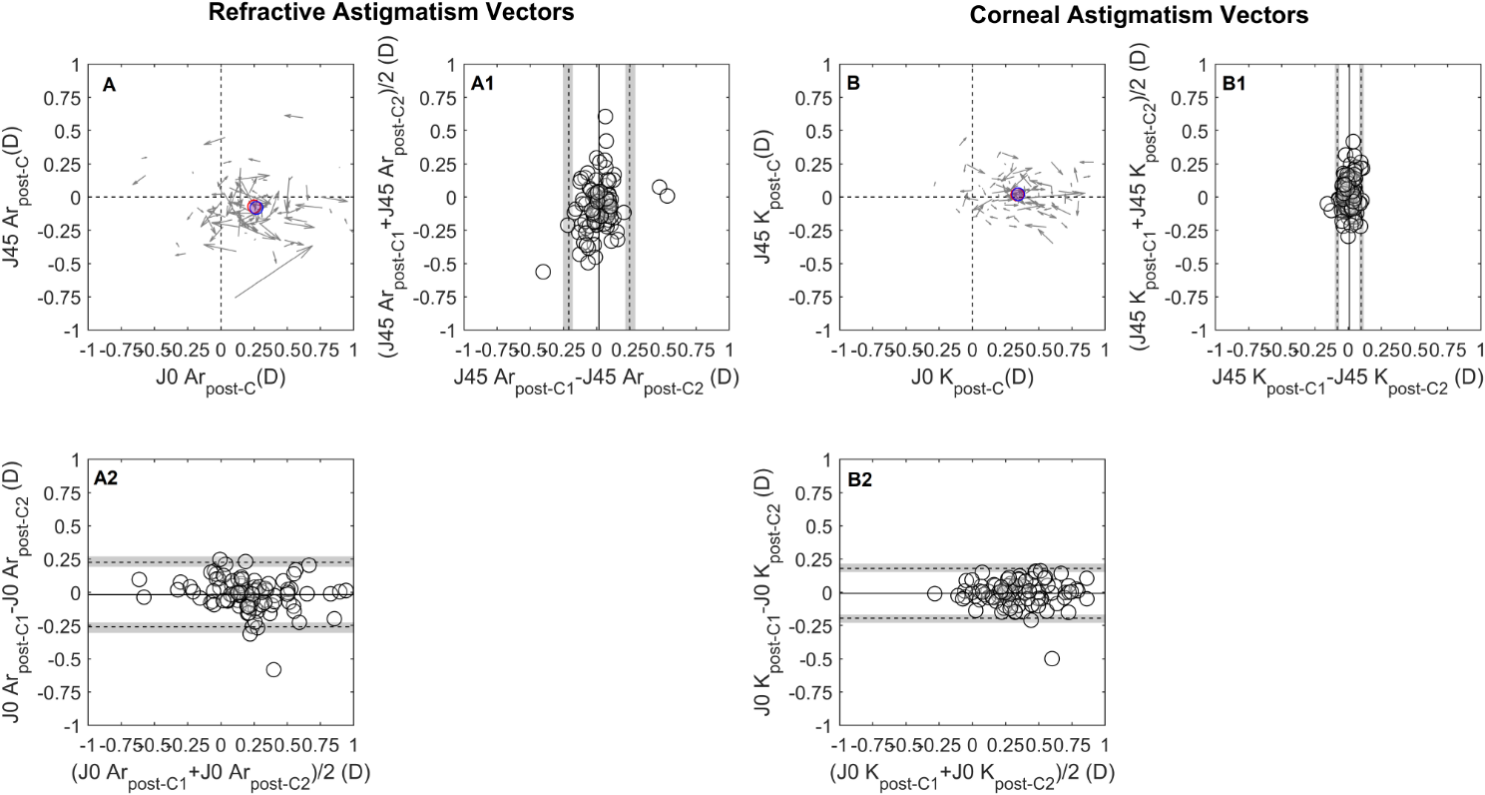
Bland-Altman (B-A) plots for the intrasession repeatability post-cycloplegia, (A) refractive and (B) corneal astigmatism vectorial components (J0/J45). The details are as shown in Fig. 2

**Fig. 4:**
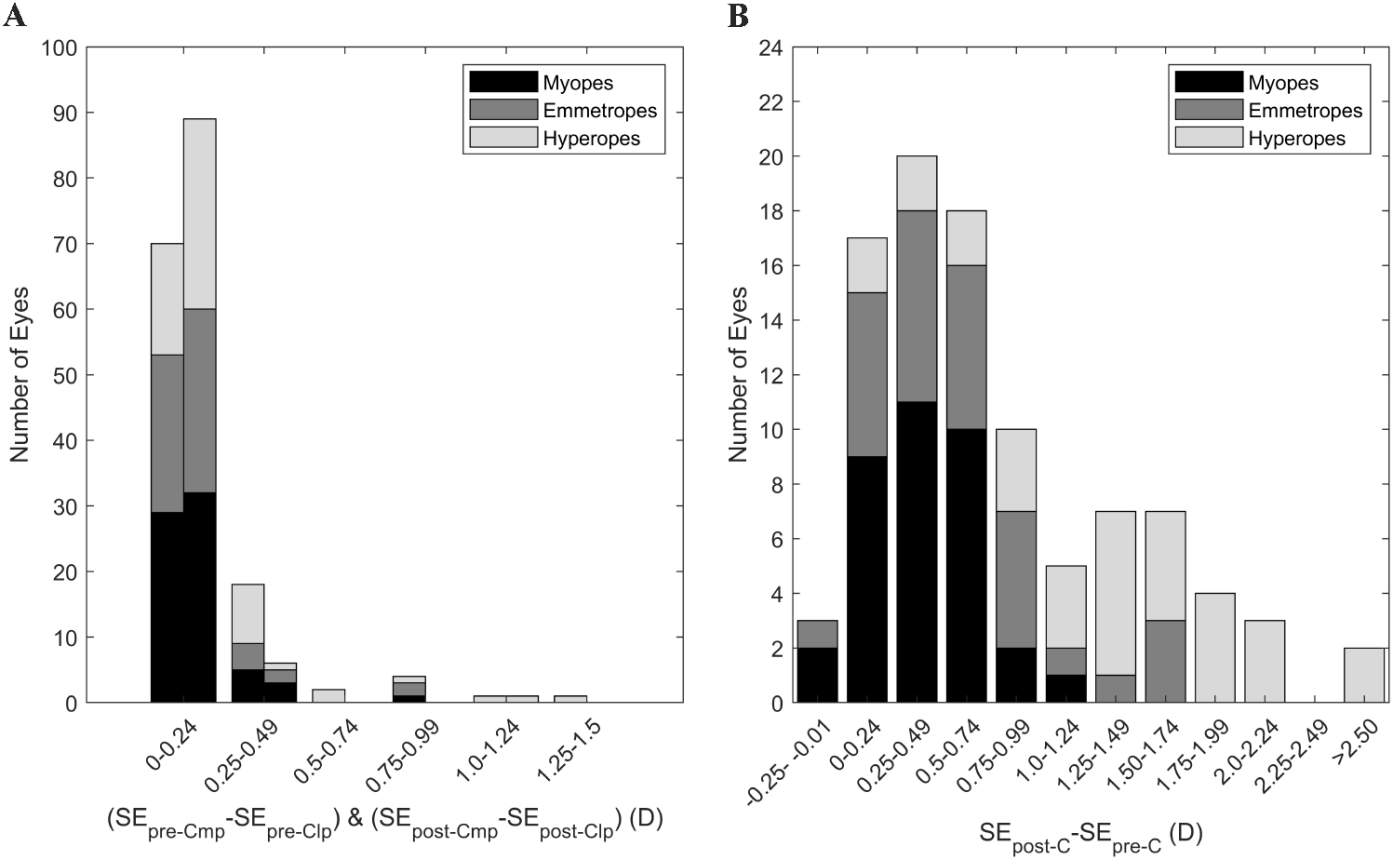
(**A**) distribution of the spherical equivalent test-retest differences both for pre-cycloplegia (first bar) and post-cycloplegia (second bar). Differences were calculated was the most positive (mp) SE minus the least positive (lp) SE. (**B**) distribution of the differences between post-cycloplegia minus pre-cycloplegia, segmented per type of refractive error

The intrasession repeatability of autorefraction showed no systematic difference or proportional bias for the SE, J0_Ar and J45_Ar. Pre-cycloplegia SE TRT was 0.65 D, improving to 0.32 D post-cycloplegia, Table 2; Fig. 1A and D. This enhancement increased the proportion of eyes within ± 0.25 D from 72.9% (CI 72.0, 73.8) pre-cycloplegia to 92.7% (CI 92.2, 93.1) post-cycloplegia, Fig. 4A. The J0_Ar and J45_Ar exhibited similar repeatability both pre- (TRT: J0_Ar = 0.25D, J45_Ar = 0.25D) and post-cycloplegia (TRT: J0_Ar = 0.25, J45_Ar = 0.19), Figs. 2A and 3A. The proportion of eyes within ± 0.12D was 82.2% (CI 81.5, 83.1) for J0_Ar and 87.5% (CI 86.2, 88.2) for J45_Ar, pre-cycloplegia; these percentages decreased to 76% (CI 75.2, 76.9) and 83.3% (CI 82.6, 84.1), post-cycloplegia.

**Table 2:** Repeatability analysis for the autorefraction and biometric parameters SE, J0_Ar, J45_Ar, Km, J0_K and J45_K and AL for pre-cycloplegia and post-cycloplegia conditions. Data are presented with mean of the differences ± standard deviation (MD ± SD), 95% CI for the mean and test-retest repeatability (TRT) with the 95% CI.

	Pre-cycloplegia		Post-cycloplegia	
Parameter	Mean ± SD 95% CI	TRT 95% CI	Mean ± SD 95% CI	TRT 95% CI
SE (D)	+ 0.01 ± 0.33 -0.06; +0.07	0.65 0.58; 0.77	+ 0.01 ± 0.16 -0.04; +0.03	0.32 0.28; 0.38
J0_Ar (D)	-0.02 ± 0.13 -0.05; +0.01	0.25 0.22; 0.29	-0.02 ± 0.13 -0.04; +0.01	0.25 0.22; 0.29
J45_Ar (D)	+ 0.01 ± 0.13 -0.02; +0.04	0.25 0.22; 0.29	+ 0.01 ± 0.10 -0.01; +0.03	0.19 0.17; 0.22
Km (D)	+ 0.01 ± 0.12 -0.02; +0.04	0.24 0.22; 0.29	+ 0.01 ± 0.14 -0.01; +0.03	0.27 0.24; 0.32
J0_K (D)	+ 0.01 ± 0.08 0.00; 0.03	0.15 0.13; 0.17	-0.01 ± 0.10 -0.03; +0.01	0.19 0.16; 0.22
J45_K (D)	0.00 ± 0.05 -0.01; +0.01	0.10 0.09; 0.12	0.00 ± 0.05 -0.01; +0.01	0.09 0.08; 0.11
AL (mm)	0.00 ± 0.07 -0.02; +0.01	0.14 0.13; 0.17	0.00 ± 0.05 -0.01; +0.01	0.09 0.08; 0.11

Pairwise Comparisons: T-test: Km, J0_K, J45_K and AL; Wilcoxon: SE, J0_Ar and J45_Ar

**Fig. 5:**
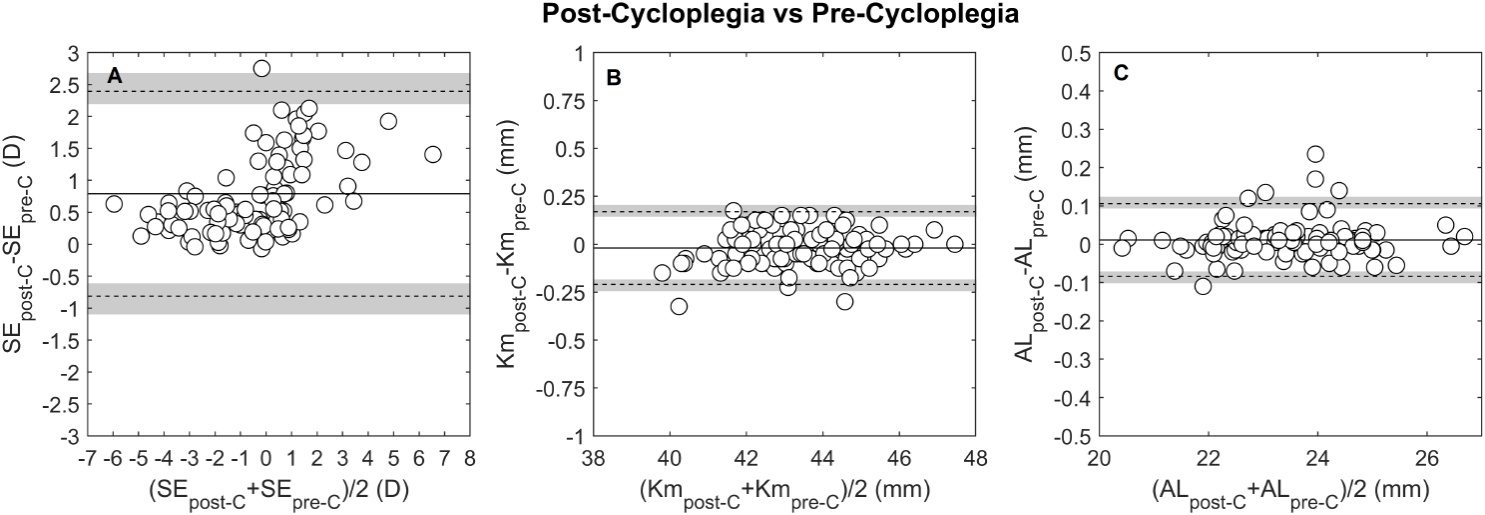
Bland-Altman plots for the agreement between pre- and post-cycloplegia measurements, for the following parameters (**A**) spherical equivalent (SE in D), (**B**) mean keratometry (Km in D) and (**C**) axial length (AL in mm). Continuous lines indicate the mean difference between post-cycloplegia and pre-cycloplegia measurement, dashed lines the 95% limits of agreement and the grey lines represent the 95% confidence interval of the limits of agreement

**Fig. 6:**
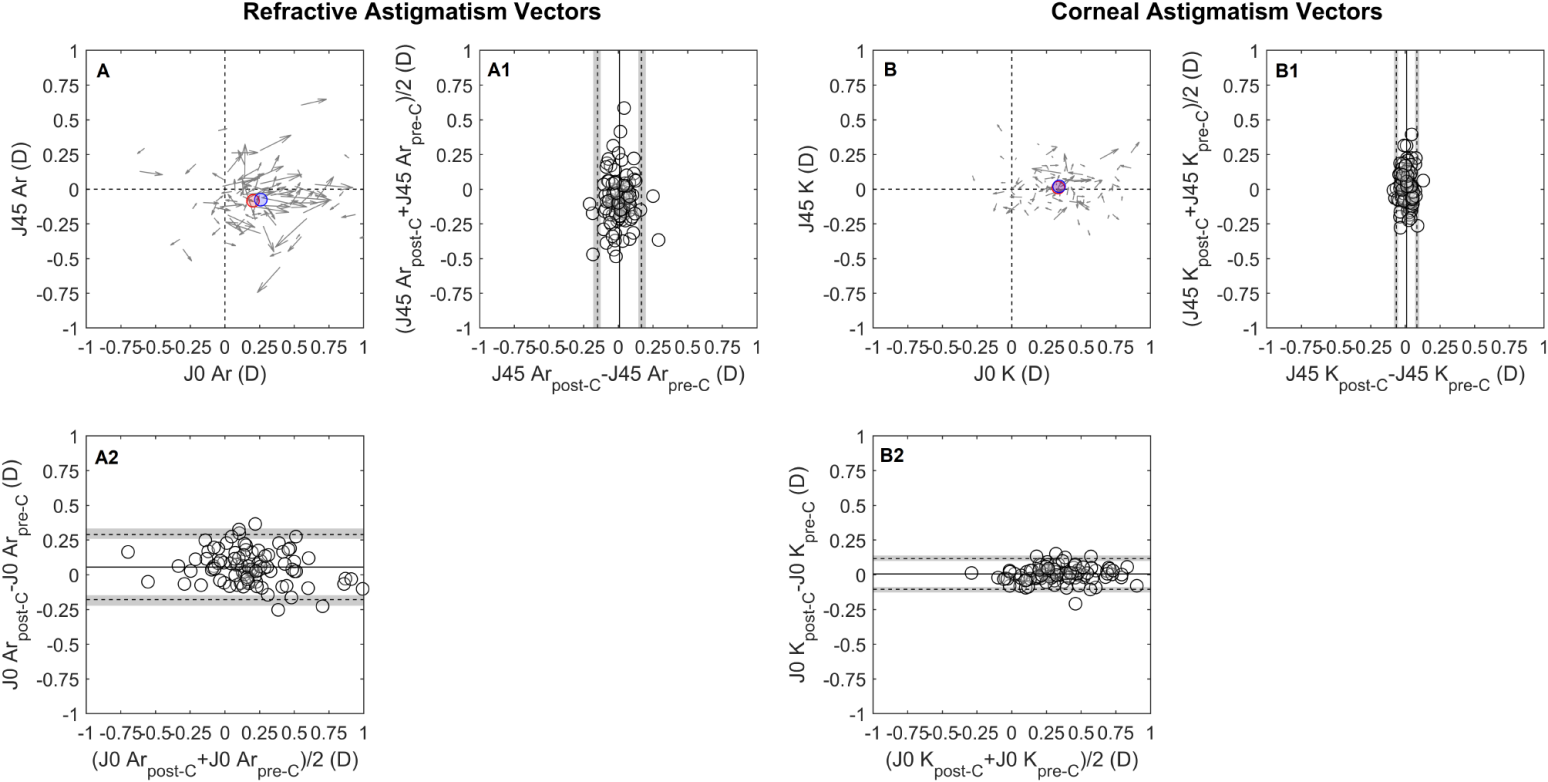
Bland-Altman (B-A) plots for the agreement between pre- and post-cycloplegia measurements, for (A) refractiveand (B) corneal astigmatism vectorial components (J0/J45). Cartesian plot shows the J0/45 difference between post-cycloplegia measurement (arrow starting point) and pre-cycloplegia measurement (arrow ending point). The details of the B-A plots are as in Fig. 2. The red and blue circles represent the mean J0/J45 coordinate pre- and post-cycloplegia, respectively

Cycloplegia influenced SE, resulting in an average increase of + 0.79D (LoA − 0.81, + 2.39) compared to pre-cycloplegia, Table 3. The proportion of eyes with differences within ± 0.25D and ± 0.50 D was 20.8% (CI 20.0 ,21.7) and 42.7% (CI 41.7, 43.7), Fig. 4B. The Bland-Altman plot, Fig. 5A; Table 4, show a proportional bias, with more hyperopic eyes eliciting higher differences between measurement conditions. The effect of cycloplegia in astigmatic components revealed a 0.06D difference (LoA − 0.18, + 0.30) in J0_Ar and no clinical difference in J45_Ar (LoA − 0.15, + 0.17), suggesting a higher manifest with-the-rule astigmatism after cycloplegia, Fig. 6A. Considering the entire group, 68.8% (CI 67.8, 69.7) of eyes had differences within ± 0.12 D for J0_Ar and 33.3% (CI 32.4, 34.3) for J45_Ar.

### Keratometry

Mean keratometry, J0_K and J45_K repeatability showed no systematic or proportional bias across the measured range. Pre-cycloplegia, the TRT for Km was 0.24 D, and post-cycloplegia, it was 0.27 D, both close to the clinically significant threshold of 0.25D, Fig. 1B and E. The percentages of eyes within ± 0.25 D were 92.7% (CI 92.2, 93.2) pre-cycloplegia and 88.5% (CI 87.9, 89.2) post-cycloplegia. For corneal astigmatic components, the J0_K TRT was 0.15 D pre-cycloplegia and 0.19D post-cycloplegia, with slightly lower values for J45_K, 0.10 D and 0.09 D, respectively, Figs. 2B and 3B. The proportion of eyes with TRT differences within ± 0.125 D was 90.6% (CI 90.0, 91.3) for J0_K and 96.9% (CI 96.5.9, 97.2) for J45_K pre-cycloplegia, and 85.0% (CI 84.7, 86.1) for J0_K and 100% for J45_K post-cycloplegia.

The agreement between keratometry readings, both pre- and post-cycloplegia, demonstrated no systematic or proportional differences, Fig. 4B. The LoA for keratometric parameters were Km (-0.21D, + 0.17D), J0_K (-0.10D, + 0.12D), and J45_K (-0.06D, + 0.08D). 98% (CI 97.7, 98.3) of the eyes displayed mean keratometry differences within ± 0.25D, and all eyes exhibited keratometric astigmatic vector differences within ± 0.125D.

**Table 3:** Agreement analysis between measurements performed pre- and post-cycloplegia for the refractive parameters SE, J0_Ar and J45_Ar; and the biometric parameters Km, J0_K and J45_K and AL. Data is presented with Mean of the differences ± SD, 95% confidence interval for the mean, limits of agreement (LoA) and 95% confidence interval for the lower and upper LoA

Parameter	Mean ± SD 95% CI	95% Lower LoA 95% CI	95% Upper LoA 95% CI	*p*-value
SE (D)	+ 0.79 ± 0.82 + 0.62; +0.96	-0.81 -1.09; -0.62	+ 2.39 + 2.20; +2.67	0.000*
J0_Ar (D)	+ 0.06 ± 0.12 + 0.03; +0.08	-0.18 -0.15; -0.22	+ 0.30 + 0.34; +0.27	0.000*
J45_Ar (D)	+ 0.01 ± 0.09 -0.13; -0.04	-0.15 -0.13; -0.18	+ 0.17 + 0.15; +0.90	0.311
Km (D)	-0.02 ± 0.10 -0.04; +0.00	-0.21 -0.24; -0.19	+ 0.17 + 0.15; +0.20	0.311
J0_K (D)	+ 0.01 ± 0.06 -0.01; +0.02	-0.10 -0.12; -0.09	+ 0.12 + 0.10; +0.14	0.279
J45_K (D)	+ 0.01 ± 0.04 0.00; +0.02	-0.06 -0.08; -0.05	+ 0.08 + 0.07; +0.10	0.017
AL (mm)	+ 0.01 ± 0.05 0.00; +0.02	-0.08 -0.10; -0.07	+ 0.11 + 0.09; +0.12	0.028

Pairwise Comparisons: T-test: J45_Ar, Km, J0_K, J45_K, AL; Wilcoxon: SE and J0_Ar

*Statistically significant differences after Bonferroni adjustment for multiple comparisons (*p* = 0.05/7 = 0.007)

### Axial length

Axial length differences between repeated measurements, both pre- and post-cycloplegia, exhibited no clinically significant disparities or proportional bias. Pre-cycloplegia, the TRT was 0.14 mm, with 88.5% (CI 87.9, 89.2) of eyes displaying differences within ± 0.1 mm. Following cycloplegia, the TRT improved to 0.09 mm, with 92.7% (CI 92.2, 93.2) of eyes exhibiting differences within ± 0.1 mm, Fig. 1C and F. The pre- and post-cycloplegia agreement indicated a mean AL difference close to zero, with the LoA ranging from + 0.10 to -0.08 mm, and 93.8% (CI 93.3, 94.2) of eyes showing differences below 0.1 mm, Fig. 4C.

**Table 4:** Repeatability and agreement analysis for the three refractive groups between measurements performed pre-cycloplegia and post-cycloplegia for the refractive parameters SE and J0_Ar. Data is presented with mean of the differences ± SD, 95% confidence interval for the mean

Parameter		Myopes	Emmetropes	Hyperopes	*p*-Value
Repeatability No Cycloplegia					
SE (D)	Mean ± SD	-0.01 ± 0.22 -0.08; +0.06 0.44 (0.35; 0.57)	-0.04 ± 0.30 -0.15; +0.07 0.59 (0.47; 0.81)	+ 0.07 ± 0.45 -0.09; +0.23 0.88 (0.71; 1.21)	-
	95% CI				
	TRT (95% CI)				
J0_Ar (D)	Mean ± SD	-0.03 ± 0.11 -0.07; +0.01 0.22 (0.18; 0.29)	+ 0.02 ± 0.12 -0.02; +0.06 0.24 (0.19; 0.32)	-0.01 ± 0.16 -0.06; +0.04 0.32 (0.26; 0.42)	-
	95% CI				
	TRT (95% CI)				
**Repeatability Cycloplegia**					
SE (D)	Mean ± SD	-0.02 ± 0.13 -0.06; +0.02 0.26 (0.21; 0.34)	+ 0.02 ± 0.12 -0.02; +0.06 0.24 (0.19; 0.32)	0.02 ± 0.22 -0.06; +0.10 0.44 (0.35; 0.59)	-
	95% CI				
	TRT (95% CI)				
J0_Ar (D)	Mean ± SD	-0.01 ± 0.12 -0.05; +0.03 0.24 (0.19; 0.31)	-0.02 ± 0.11 -0.06; +0.02 0.22(0.17; 0.30)	-0.01 ± 0.14 -0.06; +0.04 0.27 (0.22; 0.38)	-
	95% CI				
	TRT (95% CI)				
**Agreement Cycloplegia - No Cycloplegia**					
SE (D)	Mean ± SD	+ 0.38 ± 0.25 + 0.30; +0.47 + 0.87 (0.78; 1.03) -0.11 (-0.02; -0.27)	+ 0.56 ± 0.43 + 0.40; +0.72 + 1.40 (1.24; 1.71) -0.28 (-0.59; -0.12)	+ 1.47 ± 1.07 + 1.08; +1.86 + 3.56 (3.17; 4.34) -0.63 (-1.40; -0.23)	*P* < 0.001
	95% CI				
	Upper LoA				
	Lower LoA				
J0_Ar (D)	Mean ± SD	+ 0.01 ± 0.09 -0.02; 0.04 + 0.19 (0.15; 0.24) -0.17 (-0.13; -0.22)	+ 0.07 ± 0.10 + 0.04; +0.11 + 0.27 (0.34; 0.23) -0.13 (-0.09; -0.20)	+ 0.08 ± 0.15 + 0.03; +0.14 + 0.37 (0.32; 0.48) -0.21 (-0.16; -0.32)	*p* = 0.035
	95% CI				
	Upper LoA				
	Lower LoA				

Agreement multiple comparisons, only showed SE (Kruskal-Wallis) and J0_AR (ANOVA)

## Discussion

This study examined the impact of cycloplegia on the repeatability and agreement of autorefraction, keratometry, and axial length measurements in young individuals. Cycloplegia enhances autorefraction repeatability and modestly improves AL repeatability. Autorefraction repeatability is influenced by refractive error, with myopic individuals exhibiting superior repeatability compared to hyperopes. Additionally, cycloplegia induces a more positive SE, although the pre- and post-cycloplegia SE difference varies based on refractive error.

### Autorefraction

Cycloplegia significantly enhanced SE repeatability, reducing it from 0.65 to 0.31D across the entire sample, resulting in an increase in eyes with differences of up to ± 0.25 D from 73 to 93%. Among refractive error groups, emmetropes and myopes exhibited similar TRTs of ∼0.50 D, while hyperopes demonstrated higher TRT of ∼0.90 D. Despite cycloplegia halving the repeatability range to about 0.25 D, hyperopes still showed larger differences (TRT = 0.44 D) between repeated measurements, highlighting limited repeatability of the Myopia Master in hyperopic eyes. This suggests a potential necessity for additional cycloplegia (longer and higher dosage) in hyperopes for consistent autorefraction measurements.

The Myopia Master’s non-cycloplegic SE repeatability aligns with other autorefractors. For instance, Padhy et al. in a wide age-range group reported TRT values varying from 0.50 D (wavefront-based autorefractor) to 0.81D (table-top autorefractor). They linked the autorefraction repeatability to the autorefractor’s working principle [8]. Venkataraman et al. reported similar results, emphasizing enhanced repeatability in autorefractors with integrated defocusing systems [9]. In paediatric population, where high levels of accommodation are common, autorefraction repeatability may further depend on the type of autorefractor. Dahlmann-Noor et al. reported a TRT of 0.63D for a photorefraction system measuring distance at 1.0 m [17], compared to 1.57D with a hand-held system that elicits more proximal accommodation [18].

Rosenfield and Ciufreda observed improved SE repeatability following cycloplegia in a small paediatric group, particularly in instruments eliciting higher proximal accommodation. Post-cycloplegia repeatability ranged from 0.27D (retinoscopy) to 0.84D (hand-held autorefractor), in agreement with the present findings [19]. Similarly, Rauscher et al. utilizing a table-top wavefront-based autorefractometer, reported a reduction from 1.49D pre-cycloplegia to 0.64 D in the 95% interval of accommodative variations during sequential readings [20].

Cycloplegic refraction introduced a SE bias of + 0.79 D compared to non-cycloplegic refraction, attributed to a reduction in crystalline lens power [11]. In a meta-analysis, Wilson et al. observed that non-cycloplegic SE closely approximated cycloplegic SE when employing a photorefraction technique (Pluxoptix). However, hand-held and table-top autorefractors tended to underestimate hyperopia and overestimate myopia [21]. Choong et al. applying a similar cycloplegic protocol to the one in this study, reported a + 0.55 D bias with a table-top autorefractor employing optical infinity simulation fixation and a fogging system, akin to the Myopia Master [1]. Comparisons of Myopia Master autorefraction under cycloplegia with a table-top autorefractor (Nidek ARk-1) and subjective refraction revealed an average more negative SE of -0.43 D and − 0.49D respectively [4]. In contrast, to wavefront-based autorefraction, the Myopia Master exhibited a slightly more negative SE (-0.19 D), compared the Huvitz HRK8000-A and a similar SE (0.05 D) compared to the DNEye Scanner 2 [5, 6].

The bias between cycloplegic and non-cycloplegic autorefraction varies with the type of refractive error, with myopes showing the smallest difference (0.38 D), followed by emmetropes (0.56 D) and hyperopes (1.57 D). This pattern of differences aligns with findings from Fotedar et al. and Hu et al. observed in pediatric populations [22, 23]. These variations impact the Myopia Master’s sensitivity and specificity in classifying refractive errors, with potential false-negative results for hyperopes and false-positive results for myopes, affecting the detection of hyperopes and overestimate the prevalence of myopia in a population [24].

The repeatability of the pre-cycloplegia J0_Ar vector component was lower in hyperopic patients, possibly due to the greater accommodative variability and the influence of accommodation on the J0 refractive component [25]. Additionally, a subtle shift (< 0.125 D) towards with-the-rule (WTR) astigmatism was observed after cycloplegia [26, 27], driven by a decrease in the percentage of eyes with against-the-rule (ATR) astigmatism and an increase in eyes with WTR astigmatism post-cycloplegia.

### Keratometry

Cycloplegia demonstrated negligible impact on mean keratometry repeatability, with differences of 0.24 D and 0.27 D observed in pre- and post-cycloplegia measurements, respectively. These values approached the clinical significance threshold of 0.25 D. Similar trends were noted for astigmatic vectors J0_K and J45_K. Garcia-Ardoy et al. conducted a comparative study between Myopia Master keratometry and the IOL Master 700, revealing repeatability limits (0.23 D) consistent with this study, along with comparable astigmatic vector repeatability. Compared to the IOL Master 700 with a repeatability of 0.17 D, the Myopia Master keratometry exhibited slightly reduced repeatability [7].

The examination of keratometry parameters pre- and post-cycloplegia demonstrated no clinically significant differences, with agreement limits closely resembling repeatability limits. This suggests that variability in keratometric measurements pre- and post-cycloplegia aligns with that observed during repeated measurements. Regarding changes in central keratometry post-cycloplegia, these findings align with prior studies reporting no significant differences [11] or minimal clinical relevance (mean change of -0.032 ± 0.121 D) [28]. Other studies noticed corneal flattening post-cycloplegia, ranging from 0.1D [29] to 0.23 D [30]. These variations were attributed to ciliary muscle relaxation during cycloplegia, reducing tension on the scleral spur and resulting in peripheral corneal curvature flattening, exerting only a minor influence on central keratometry [29].

### Axial length

Cycloplegia marginally improved AL measurement repeatability (∼1.5x), narrowing the limits from ± 0.14 mm to ± 0.09 mm. This repeatability is comparable to that reported for the Myopia Master in a group of myopic individuals (TRT: 0.11 mm) [7]. Sheng et al. using partial coherence interferometry (IOLMaster), the same biometric principle used by the Myopia Master, reported enhanced post-cycloplegia AL repeatability (TRT: 0.07 mm) compared to pre-cycloplegia (TRT: 0.09 mm) [31]. This improvement may be attributed to the ciliary muscle’s paralyzing effect which reduces crystalline lens thickness fluctuations, enabling a more stable axial length calculation [12].

Although no association between SE differences and AL differences was observed in the present data, a minor elongation of 0.01 mm with LoA near 0.1 mm was noted with cycloplegia. These findings align with previously reported differences ranging from 0.0 to 0.013 mm [11, 28, 31]. Cheng & Hsieh proposed that the observed 0.013 mm elongation observed in their study was associated with a posterior movement of the lens body, compressing the vitreous humour and, subsequently, elongating the AL [28].

Assuming a 0.1 mm variation in AL corresponds to a dioptric change of 0.25D, the repeatability of axial length measurement under cycloplegia enables refractive monitoring below the clinical threshold of 0.25 D. Moreover, since AL repeatability is independent of the AL magnitude, unlike SE measurements, AL measurement may be the most reliable method for monitoring refractive error progression, particularly if performed under cicloplegia.

## Conclusions

This study underscores the efficacy of cycloplegia in enhancing refraction repeatability. Cycloplegia effectively narrows the variability of repeated measurements within a 0.25 D range, particularly demonstrating a significant reduction in hyperopic eyes. Notably, AL measurements under cycloplegia emerge as the least variable measure in monitoring refractive error.

## Data Availability

The datasets analysed during the current study are not publicly available its disclosure in public databases is not covered by the proposal approved by the ethics committee but are available from the corresponding author on reasonable request.

## Abbreviations

AL: Axial length
ANOVA: Analysis of variance
CI: Confidence interval
DCVA: Distance corrected visual acuity
Km: Mean keratometry
LoA: Limits of agreement
MD: Mean difference
SD: Standard deviation
SE: Spherical equivalent
TRT: Test-Retest repeatability
